# Review of antimicrobial administration in cases of proven bacteraemia in adult patients at one London teaching hospital

**DOI:** 10.1186/2197-425X-3-S1-A406

**Published:** 2015-10-01

**Authors:** A Myers, J Hatcher

**Affiliations:** King's College Hospital, London, United Kingdom; Department of Microbiology, Imperial College, London, United Kingdom

## Introduction

The death of 12 year old Rory Staunton led to a U.S. Senate Hearing on Sepsis (Sept 2013). Rory's Regulations (part of New York State Law) state that evidence-based protocols must be used in identification and management of sepsis. In the UK, practice is guided by the Surviving Sepsis campaign [1]. Timely and appropriate antibiotic administration is of fundamental importance [2].

## Objectives

1) To assess when septic patients receive antibiotics in a London teaching hospital. A subset of septic patients were reviewed: those with proven bacteraemia.

2) Are appropriate antimicrobials administered?

## Methods

A retrospective review of bacteraemic adults with sepsis was carried out at St Mary's Hospital during May, June and July 2014. Data was obtained from patient notes, observation and drug charts, individual patients, the medical teams, and the microbiology laboratory. Record was made of demographics, time of presentation with sepsis, time of recognition, timing and choice of antimicrobial, and outcomes.

## Results

42 cases were reviewed. 13 presented with severe sepsis and 4 out of the 42 eventually died of sepsis. A third received antibiotics within an hour and a third in 2-3hrs. 7 patients waited more than 9hrs, 3 of whom died. *E.coli* was the responsible organism in 45% of cases, Staphylococci accounted for 19% and Streptococci 12%. In 75%, initial suspicion of source was correct. Antimicrobials were prescribed according to protocol or microbiology advice in 83%. Antibiotic therapy was altered following pathogen identification in 55% of cases. Resistance mechanisms were present in 15% of gram negatives (AMP C or ESBL). Reasons for delay in receiving antimicrobials included: late recognition; complex presentation; antibiotics not administered at the time of prescription; no stat dose prescribed.

## Conclusions

Most cases were recognised and treated appropriately within 3 hours. Some, however, had an unacceptably long delay. Most patients survived their bacteraemia and did not require ICU admission. Ward doctors are generally good at assessing the likely source of sepsis and most use guidelines or specialist advice to guide therapy. Management could be improved by encouraging doctors to administer antibiotics personally at the time of assessment and to measure lactate, especially when presentation is atypical. Most organisms are sensitive and unnecessary broadening of empiric antibiotic therapy beyond guidelines is not required. However, due to a significant minority of resistant pathogens, close liaison with Microbiology is recommended.
